# Defining the Optimal Strategies for Achieving Drug-Free Remission in Rheumatoid Arthritis: A Narrative Review

**DOI:** 10.3390/healthcare9121726

**Published:** 2021-12-13

**Authors:** Hanna Gul, Kate Harnden, Benazir Saleem

**Affiliations:** 1 Leeds Institute of Rheumatic and Musculoskeletal Medicine, 2nd Floor, Chapel Allerton Hospital, Chapeltown Road, Leeds LS7 4SA, UK; kateharnden@doctors.org.uk; 2 NIHR Leeds Biomedical Research Unit, Chapel Allerton Hospital, Chapeltown Road, Leeds LS7 4SA, UK; benazir.saleem@nhs.net

**Keywords:** rheumatoid arthritis, remission, drug-free remission, b-DMARDs, cs-DMARDs, tapering

## Abstract

*Background:* It is now accepted that the optimum treatment goal for rheumatoid arthritis (RA) is sustained remission, as this has been shown to be associated with the best patient outcomes. There is little guidance on how to manage patients once remission is achieved; however, it is recommended that patients can taper therapy, with a view to discontinuing and achieving drug-free remission if treatment goals are maintained. This narrative review aims to present the current literature on drug-free remission in rheumatoid arthritis, with a view to identifying which strategies are best for disease-modifying anti-rheumatic drug (DMARD) tapering and to highlight areas of unmet clinical need. *Methods:* We performed a narrative review of the literature, which included research articles, meta-analyses and review papers. The key search terms included were rheumatoid arthritis, remission, drug-free remission, b-DMARDS/biologics, cs-DMARDS and tapering. The databases that were searched included PubMed and Google Scholar. For each article, the reference section of the paper was reviewed to find additional relevant articles. *Results:* It has been demonstrated that DFR is possible in a proportion of RA patients achieving clinically defined remission (both on cs and b-DMARDS). Immunological, imaging and clinical associations with/predictors of DFR have all been identified, including the presence of autoantibodies, absence of Power Doppler (PD) signal on ultrasound (US), lower disease activity according to composite scores of disease activity and lower patient-reported outcome scores (PROs) at treatment cessation. *Conclusions:* DFR in RA may be an achievable goal in certain patients. This carries importance in reducing medication-induced side-effects and potential toxicity, the burden of taking treatment if not required and cost effectiveness, specifically for biologic therapy. Prospective studies of objective biomarkers will help facilitate the prediction of successful treatment discontinuation.

## 1. Introduction

Rheumatoid arthritis (RA) is a chronic immune-mediated systemic disease, which affects approximately 1% of the population and is characterized by a symmetrical inflammatory polyarthropathy [1].

Over recent years, there has been a paradigm shift in the treatment approach in RA from cautious escalation of therapies for symptomatic relief to the early and rapid control of inflammation soon after diagnosis, aimed to prevent structural damage and preserve function. This is in accordance with the ‘window of opportunity’ hypothesis, which suggests that in early RA, aggressive treatment can reverse underlying autoimmunity and induce immune tolerance (thus potentially modifying the disease course) [2]. In clinical practice, this is achieved using a treat-to-target (T2T) strategy. This strategy involves strict monitoring of disease activity using composite measures, e.g., disease activity score (DAS28) resulting in successive escalation of immunosuppressive agents (conventional synthetic and biologic disease-modifying drugs (cs-DMARDs and b-DMARDs, respectively). These drugs are used alone or in combination and with or without corticosteroids to control inflammation [3].

This shift in treatment approach has led to increasing numbers of patients achieving remission, with dramatically improved outcomes. Thus, ongoing management of RA should be focused on maintaining this (European League Against Rheumatism (EULAR) treatment recommendations update 2019) [4]. The potential to achieve drug-free remission (DFR) is also very important. Chronic immunosuppressive therapy, particularly b-DMARDs, can be associated with adverse events, which include a dose-dependent increased risk of infections and malignancy [5]. They are also expensive, costing in excess of GBP 6k pa, per patient in the UK [6]. Thus, tapering (reducing treatment with the long-term aim of stopping, whilst maintaining treatment goals) of b-DMARDs in patients in remission is a key management issue [7].

Despite being recognized as an important treatment goal, there is little guidance on how to manage remission once it has been achieved. This is because existing studies are largely heterogeneous with respect to the way they define remission and flare and due to differences in populations studied, e.g., established vs. early RA [8]. Furthermore, there is no uniform definition of sustained remission [7]. Despite achieving remission, it has been demonstrated that a proportion of patients can progress radiographically. This is thought to be due to the use of composite clinical measures to define remission in clinical practice and trials, which are largely subjective. Notwithstanding these challenges, up to 50% of patients can achieve sustained clinical remission (≥6 months) following treatment [3,8].

Experience with b-DMARD tapering is largely with tumor necrosis factor inhibitors (TNFis). Tapering of cs-DMARDs, notably methotrexate (MTX), is also desirable for patients concerned about long-term side-effects and the burden of taking tablets/self-injecting if they are well [9,10]. These frequently lead to poor treatment compliance, with approximately 15% of patients self-discontinuing treatment, which itself can lead to increased disease morbidity [10,11].

International guidance (notably EULAR [4] and the American College of Rheumatology (ACR) [12]) do recommend tapering of treatment after the achievement of remission and they advise a specific sequence of reduction, based on cost and effectiveness (starting with corticosteroids, b-DMARDs, and then cs-DMARDs). However, despite these recommendations, there remains a lack of consensus of how to deliver this approach. Some data show that tapering is feasible in a proportion of RA patients that achieve remission; however, the ideal patient profile is unknown. A small (3.6 to 22 % prevalence) but not inconsiderable number of patients with RA may have a chance for DFR, the closest state to RA cure.

This narrative review aims to present the current literature on DFR in RA, with a view to identifying which strategies are best for DMARD tapering and to highlight areas of unmet clinical need.

## 2. Defining Remission in RA

To be able to identify individuals who are more likely to achieve DFR, we first need to be able to define remission accurately. Remission in RA is currently defined clinically using a cut-off of the DAS28 (disease activity score). It incorporates a mathematical formula comprising the number of tender and swollen joints out of 28 (TJC28, SJC28), a serum marker of inflammation (e.g., C-reactive protein, CRP) and an optional measure of patients’ assessment of global health status (PGA) [13].

DAS28-remission has been defined as a score of <2.6 [14,15]. It is the standard measure used in clinical practice; however, it is not a precise assessment of remission. This score and tender joint count assessment may be influenced by physical comorbidities, e.g., osteoarthritis or psychosocial factors. Swollen joint counts may also be inaccurate in remission [16], while objective serological inflammatory markers (ESR and CRP) are non-specific to RA. Furthermore, the DAS28 joint count excludes the feet and ankles, therefore missing active disease in these areas [11]. It has been shown that some patients in remission do still have evidence of subclinical synovitis on musculoskeletal ultrasound (US) [17,18,19,20].

There have been multiple attempts to define clinical remission more stringently, including the ACR/EULAR 2011 Boolean remission criteria (TJC28, SCJ28, CRP and PGA all ≤1) [21,22], CDAI (TJC + SJC + PGA + Physician GA: remission = 0.0–2.8) [23] and SDAI (TJC + SJC + PGA + Physician GA + CRP: remission is ≤5) [24] scores (comprehensive and simplified disease activity scores, respectively); however, these still include subjective measures and potentially inaccurate joint counts [21,24]. The concept of ‘deep’ clinical remission has been considered (DAS28 < 1.98), which is suggested to reflect the absence of biological inflammation; however, longitudinal outcome data relating to this target have not yet been studied prospectively [25].

Physical examination is known to have a low sensitivity for the detection of mild synovitis, such as that found in clinical remission; however, musculoskeletal US has proven to be an excellent tool to identify subclinical inflammation that is associated with risk of relapse and structural damage [26,27,28]. Despite this, the definition of what constitutes imaging remission remains challenging [19,28,29]. More recently, immunological status has been shown to predict the likelihood of sustained remission in RA [30,31]. This adds another potential dimension to consider when defining the remission state in RA.

**Figure 1:**
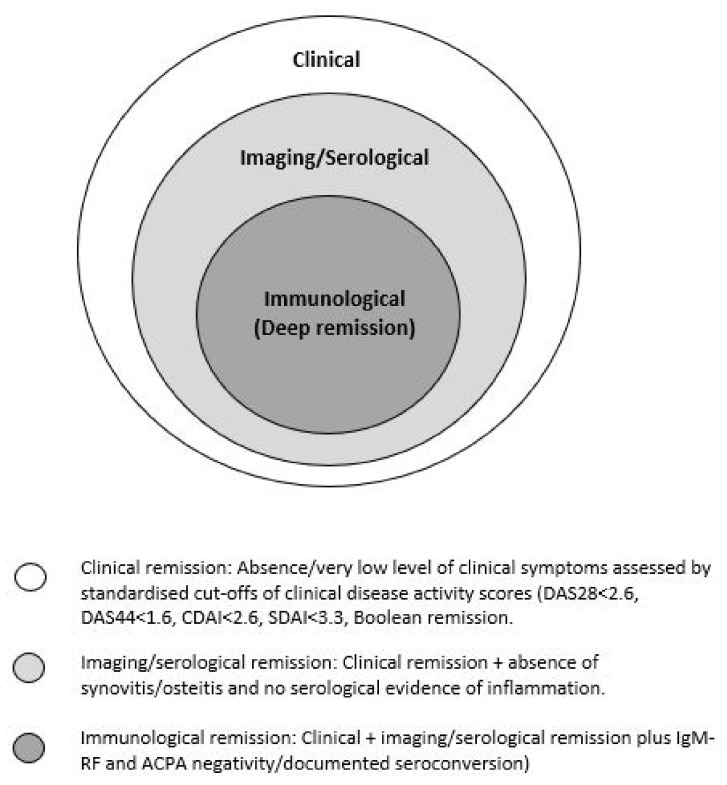
Shell model of remission states adapted from Schett et al. [7].

Schett et al. [7] have recently introduced the concept of ‘multi-level’ remission aimed to characterize remission more precisely (Figure 1). It involves the achievement of different levels/depths of remission. It suggests that a state of deep remission may be attained if all three categories are achieved; however, this has not yet been used prospectively.

## 3. DFR Remission in Patients with RA Treated with cs-DMARDs

DMARDs are indicated for the treatment of inflammatory arthritis, e.g., RA; however, they are also used to treat other disorders [32]. cs-DMARDs are typically used as first-line agents, alone or in combination. Commonly used cs-DMARDs include methotrexate (MTX), hydroxychloroquine (HCQ), leflunomide (LEF) and sulfasalazine (SSZ). They are mostly oral preparations (except for MTX, which can also be injected subcutaneously) [33].

Some of the earliest data on withdrawing cs-DMARDS come from historical observational studies. These studies often focus on older conventional cs-DMARDs, which are no longer used in first-line RA treatment, e.g., gold and d-penicillamine [34,35]. It has been demonstrated that DFR is possible in a minority of cases. Most of the evidence for discontinuing cs-DMARDs to achieve sustained DFR comes from randomized controlled trials (RCTs) for patients with stable RA on a range of monotherapies [34,36,37,38,39]. Many of the DMARDs studied, however, are now rarely used in practice. Additional evidence comes from RCTs and observational studies in which a step-down approach in treatment was followed (combination DMARDs reduced to monotherapy). These demonstrated sustained clinical response to treatment after tapering in early RA patients [40,41,42,43].

Table 1 summarizes the studies discussed.

In a small, 15-year observational study, Tiippana-Kinnunen et al. [44] used a ‘sawtooth strategy’ to discontinue cs-DMARDs. Overall, cs-DMARDS were discontinued in 29% of patients due to remission or low disease activity. Of note, 45% of these patients had a disease flare, some several years after treatment discontinuation. Of the patients that did have to restart cs-DMARD therapy, none were in clinical remission after 15 years.

In a review of two large RA cohorts, sustained cs-DMARD-free remission was found to occur in 15% of patients in the Leiden Early Arthritis Clinic cohort and 9.4% in the British Early Rheumatoid Arthritis Cohort (EAC) [45].

More recently, the KIMERA trial [46] included 234 patients that discontinued cs-DMARDs. Overall, 50 patients discontinued their cs-DMARDs but 31 (62%) experienced a flare after stopping. DFR was maintained at 48 months in 46.1% of patients achieving remission.

In the Biomarkers of Remission in Rheumatoid Arthritis (BioRRA) Study [47], established RA patients deemed to be in clinical and ultrasound remission discontinued their DMARDs and were monitored for six months. Twenty-one out of 44 (48%) patients achieved DFR at the end of the study.

One of the earliest RCTs by ten Wolde et al. [36] randomized 142 RA patients to continue therapy and 143 received a placebo. Over 52 weeks, flare occurred in 22% of patients who continued their cs-DMARD but occurred in 38% of patients who received a placebo. One limitation of this study was that it did not include many patients on methotrexate; therefore, the benefits of remaining on cs-DMARDS may have been underestimated.

Ahern et al. found that attempting gradual d-penicillamine withdrawal caused 80% of patients to flare [34]. Further RCTs that have looked at cs-DMARDs continuation have found that patients who have received a placebo clinically deteriorated compared to the patients that continued cs-DMARD therapy [37,38,39].

In a meta-analysis of six RCTs published before 2000, it was shown that withdrawing cs-DMARD therapy resulted in a significantly higher risk of flare (46%) compared to those that remained on treatment (17%). A total of 501 RA patients that were included had a disease duration that ranged between 40 months to 16 years. Limitations of the studies included the use of cs-DMARDs that are rarely used and the lack of patients with early disease [48].

The BeST study [49], a multi-center randomized single-blind trial, used tightly controlled targeted treatment strategies with the aim of achieving remission in 508 RA patients. All patients included had active but early disease with a symptom duration <2 years. The patients were randomized into four different treatment groups. Group 1: sequential monotherapy with methotrexate, group 2: step up combination therapy with other cs-DMARDs, group 3: combination therapy with methotrexate, sulfasalazine and high-dose tapered oral corticosteroid (prednisone), and group 4: combination therapy with MTX and infliximab (IFX). The patients were followed over 7 years of targeted treatment and DAS scores were measured every 3 months. After 2 years, if their DAS44 was <1.6 for at least half a year, the DMARD was tapered and discontinued. After 5 years, 14% were in DFR. Over the 5 years, 23% of patients achieved DFR at some point but of the 46% who lost remission, 74% re-gained remission after 3–6 months of restarting treatment. After 7 years, 15% of patients were in DFR and further analysis after 10 years showed that 14% were in DFR. Of these 14%, there were no differences among the T2T strategies.

The tREACH trial [50] randomized 281 early RA patients to start initial treatment with triple cs-DMARD therapy (MTX, SSZ and HCQ) with glucocorticoid bridging or MTX monotherapy with glucocorticoid bridging. The patients were monitored every 3 months and were switched to a TNFi and MTX if the DAS28 was >2.4. If the DAS28 was <1.6 at two consecutive timepoints, the DMARDs were tapered according to study protocol. Tapering was initiated in 118 patients receiving cs-DMARDS and 41% flared within a year, although 65% regained remission within 6 months. Of the 34 patients achieving DFR, 7 patients remained in remission after 6 months.

The IMPROVED study [51] demonstrated that patients who achieve early remission, within two years, more often achieve DFR. Patients were all started on high-dose prednisolone (60 mg) and methotrexate that was quickly escalated up to 25 mg. After 4 months, 387 (63%) patients achieved remission, and their medications were tapered and stopped. The prednisolone was tapered after 4 months and the MTX after 8 months. Thirty-two percent of the patients who achieved early remission at 4 months were able to go on to achieve DFR and 29% remained in drug-free remission after 2 years.

## 4. Predicting DFR for Patients Receiving Treatment with cs-DMARDs

Several factors have been demonstrated to predict the successful maintenance of remission after cs-DMARD withdrawal to achieve DFR. cs-DMARD-free remission is more likely to be achieved when T2T strategies have been employed with the goal of establishing remission earlier in the RA disease course. This supports the window of opportunity hypothesis for RA treatment [2]. In addition, other factors associated with cs-DMARD-free remission include a longer duration of sustained remission prior to drug withdrawal [19], the absence of autoantibodies (ACPA and RF) [36,45,52] and lower disease activity (DAS28 < 2.6) at the time of treatment cessation [45,47,52,53]. Using methotrexate as the last cs-DMARD before withdrawal has also been associated with a higher chance of achieving DFR [52,54].

The BioRRA study [47] is the most comprehensive study of biomarkers for predicting cs-DMARD remission to date. Baker et al. developed a composite score for the prediction of DFR including circulating inflammatory biomarkers, and peripheral CD4+ T-cell gene expression. This score was able to differentiate future flare from DFR with an AUROC (receiver–operator characteristic) of 0.96 (95% CI 0.91–1.00), sensitivity 0.91 (0.78–1.00) and specificity 0.95 (0.84–1.00). Limitations of the study include small patient numbers and the heterogeneity of cs-DMARDs included. Ultrasound biomarkers were not identified.

Similarly, Gul et al. [55] aimed to assess the rate of sustained remission over 12 months (without flare) for RA patients in stable remission and to evaluate associated factors, with a view to developing a predictive model for successful tapering of cs-DMARDs. They conducted a prospective observational study of 200 RA patients in DAS28 remission who were offered either tapering or continuation of their cs-DMARD. Of those who tapered, 64% remained in clinical remission after 12 months compared with 80% of patients on stable treatment. In the tapering group, higher CRP, TJC, an inflammation-related T-cell (IRC) and PROs were associated with flare (all *p* < 0.05), with a trend for higher total PD (*p* = 0.066), which contradicts the findings of the BioRRA study [47]. A model predicting sustained remission retained RAQoL (RA Quality of Life score), total PD score and %IRC (85% accuracy, AUROC = 0.893, *p* < 0.0001).

Overall, it has been shown that cs-DMARD-free remission can occur in 14–48% of patients achieving remission, although discontinuing cs-DMARDS carries a 38–80% risk of disease flare. This is probably unacceptably high for both patients and clinicians alike due to the negative impact of flare on QoL [56] and the risk of disease progression [49]; however, it has been reassuringly demonstrated that most patients can re-capture remission following treatment for flare. Identifying patients who can achieve successful withdrawal of cs-DMARDs remains an area of unmet clinical need, despite reports of potential predictive factors in the literature. However, progress in this field is promising.

## 5. DFR Remission in Patients with RA Treated with Biological Therapies (b-DMARDs)

b-DMARDs can target and inhibit specific pathways of the immune system and inflammatory cascade, each with a unique mechanism of action. TNFis include: etanercept (ETN), adalimumab (ADA), infliximab (IFX), golimumab (GOL) and certolizumab-pegol (CZP). Other agents include rituximab (RTX, anti-CD19 agent), abatacept (ABA, humanized fusion antibody), tocilizumab (TCZ, anti-IL6) and small molecule Janus Kinase (JAK) inhibitors, e.g., baricitinib/tofacitinib, amongst others [32].

Several studies have analyzed the effects of b-DMARD (mainly TNFi) withdrawal in RA patients after a successful remission induction regime and will be discussed. The remission induction regime varies between studies involving different drugs. Furthermore, there is inconsistency in the definitions of remission used; duration of remission; and the duration RA: from DMARD naïve to established RA. Often, drug tapering or successful dose reduction may be the primary outcome. For the purpose of this review, only studies evaluating DFR remission will be discussed. To date, there is no evidence in the literature for discontinuation of RTX or JAK inhibitors.

Table 2 summarizes the studies discussed.

### 5.1. DFR Following Remission Induction with TNFi Therapy

The foremost b-DMARD withdrawal study involved 20 treatment-naïve RA patients in a double blind RCT [57]. As well as demonstrating superior clinical outcomes after 1 year of treatment with IFX and MTX compared to MTX monotherapy, 70% of patients were able to sustain the response 12 months after therapy cessation. However, these rates have not been reproduced in other IFX withdrawal studies.

The BeSt study [58,59] was unique at the time of design in that it focused on sustained remission and de-escalation of therapy (described previously). The fourth treatment arm involved the use of IFX and MTX as remission induction agents [57]. During the study period, 77 out of 120 patients were able to discontinue IFX and 66/77 (56%) were able to permanently discontinue therapy during the 2-year follow-up. However, as previously mentioned, the longer-term (7 year) follow-up of this study revealed no difference in the percentages of patients in DFR between the groups and that an important factor in predicting DFR was an early and strict DAS-targeted management plan.

The IDEA study [60] compared remission induction with IFX and MTX versus MTX and high-dose methylprednisolone for patients with early RA, using a similar DAS-targeted management plan to the BeST study. If DAS44 remission was achieved at week 26, IFX was stopped. It was found that IFX was not clinically superior to high-dose steroids with similar remission rates; however, of the IFX group, 25% (14/55) achieved sustained remission after stopping IFX.

In the RRR study, IFX was stopped in RA patients with persistent low disease activity (LDA). Interestingly, 55% of the patients remained in the low disease activity/remission status for at least one year, despite stopping TNFi treatment [61].

ADA-free remission is possible in patients with established RA (HONOR STUDY) [62] but more likely in patients with early, MTX-naïve RA (OPTIMA Study) [63]; remission rates were 21% vs. 66 % at 52 weeks, respectively.

In 2015, Smolen et al. [64] randomized patients with RA 1:1 to CZP or placebo plus current cs-DMARDs. At week 24, patients who achieved the primary endpoint of CDAI remission at both weeks 20 and 24 stopped study treatment and continued in the study until week 52. The authors concluded that remission could not be maintained after withdrawal of CZP.

The next two studies (PRIZE [65] and PredictRA [66]) aimed to determine if dose reduction was superior to cessation in maintaining remission, although there are other studies that are consistent with the findings described below (DOSERA [67], DRESS [68], STRASS [69], RETRO [70], AGREE [71]). In the PRIZE study [65], the potential of MTX plus ETN to achieve remission was addressed in early RA. In this study, more than 60% of the patients achieved remission. Those patients achieving remission were then randomized into three strategy arms, which involved tapering of ETN, stopping it or stopping both MTX and ETN. Remission rates after 1 year were 62%, 40% and 23%, respectively, showing that the level of reduction in treatment was associated with the relapse rate in patients. While more than half of the patients-maintained remission while tapering, withdrawal of ETN was possible in less than half of the patients and complete withdrawal of DMARDs only in one quarter of the patients.

PredictRA [66] aimed to investigate the association between baseline disease activity and the occurrence of flares after ADA tapering or withdrawal in patients with established rheumatoid arthritis (RA) in sustained remission. In this double-blind, randomized trial, patients in remission for >6 months on ADA were recruited and, after a 4-week open-label period, were randomized to taper to 3 weekly ADA or cessation. A lower percentage of patients in the taper arm (37, 36%) than in the withdrawal arm (9, 45%) experienced a flare by week 40. Of particular interest, there were no objective MRI imaging predictors of flare and a significant proportion of patients that flared did not regain response, despite restarting therapy. This may be a representative of the cohort who had longstanding RA.

The studies discussed so far suggest that DFR remission post-TNFi therapy is possible and that there may be a trend toward a more successful outcome with dose tapering versus complete cessation. Conclusions do, however, need to take into account the heterogenicity of the studies available. An important question is whether improved selection of remission patients could yield higher rates of sustained remission after cessation of therapy?

Previous studies have suggested that DFR is only viable in patients with early disease and deeper or longer clinical remission who are more likely to be able to successfully withdraw from therapy [7] (further discussed in Section 6). The benefit of early treatment on successful cessation of TNFi was confirmed in a study involving patients (n = 47) who discontinued TNFi and found that DFR occurred more often in patients starting TNFi early in their disease course (59%) compared to patients starting TNFi late in their course of disease (15%) [72]. Based on this concept, the EMPIRE study assessed the efficacy of ETN plus MTX versus MTX monotherapy in patients with very early inflammatory arthritis. Therapy was stopped at 26 weeks if the patient had no tender or swollen joints and at 52 weeks, all patients stopped ETN and MTX. However, this regime of early treatment with ETN did not increase the chance of DFR [73].

Recently, in the 2-year results of the TARA study [74], van Mulligen et al. aimed to assess the effectiveness of two different tapering strategies after 24 months: (i) tapering of cs-DMARD first (MTX in approx. 90% of patients), followed by TNFi and (ii) tapering the TNFi first, followed by cs-DMARD. Out of 189 patients, 61% of patients flared at 2 years and DFR was achieved in only 15% (slightly more frequent if following the first tapering strategy). They concluded that the order of tapering did not affect flare rate, although from a financial perspective, tapering the TNFi first is likely more favorable (in-line with EULAR recommendations) [4].

### 5.2. DFR Following Remission Induction with Abatacept (ABA) Therapy

In the AVERT trial [75], patients with low disease activity at month 12 entered a 12-month period of withdrawal of all RA therapy. While 61% of the patients reached remission with ABA plus MTX, only 15% of the patients-maintained remission for 12 months after discontinuing ABA. The high relapse rate in this study may be attributed to the fact that MTX was concomitantly stopped and, even more importantly, that a sustained remission was not ensured before abatacept was stopped; hence, stopping of abatacept may have been initiated too early.

### 5.3. DFR Following Remission Induction with Tocilizumab (TCZ) Therapy

The DREAM study investigated the cessation of TCZ treatment in 187 rheumatoid arthritis patients with established disease who achieved remission following TCZ therapy [76]. At 12 months follow up, only 9% of patients were still in remission.

The ACT-RAY [77] study withdrew TCZ after sustained remission. The 2-year study results revealed that 50.4% discontinued TCZ after achieving sustained remission and only 5.9% achieved drug-free remission. This study also employed a T2T approach during the initial treatment phase of the study.

## 6. Predictors of DFR for Patients Receiving Treatment with b-DMARDs

### 6.1. Clinical and Demographic Variables

Although several potential biomarkers of b-DMARD-free remission have been reported, validated measures are yet to be identified. Shorter disease duration [8,62,72,78,79], fewer or absence of erosions [71,77] and low disease activity at baseline [8,59,61,70,80,81] have been consistently associated with successful discontinuation in several studies.

There is potential reversibility of autoimmunity in early disease. Subsequently, remission induction during this phase can increase the chance of successful b-DMARD discontinuation. This reversibility decreases with time, following which chronic synovitis ensues, in addition to persistent cytokine abnormalities, which can lead to structural progression. Thus, the efficacy of treatment may be reduced for patients with longer disease duration, resulting in only moderate clinical benefit and a reduced chance for DFR [82]. This concept is supported by the observation that treatment responses in the first 3 months following diagnosis can predict the later achievement of remission [83].

Persistent autoimmunity drives inflammation; therefore, serum markers of inflammation have also been studied as predictors of DFR in RA. Although measures of CRP and ESR are widely used to assess inflammation in RA, these are non-specific and can be raised due to other co-morbidities, including obesity. Importantly, they do not assess local inflammatory activity and related processes, i.e., structural damage at the joint level [7].

The concept of ‘deep’ clinical remission has been explored by several studies in order to determine what extent of remission provides the greatest predictability of sustained remission following tapering or discontinuation of TNFis. A baseline DAS28 of <2.22 and <1.98 was found to be associated with sustained DFR in the RRR [61] and HONOR [62] studies, respectively. In the ANSWER Cohort study, Boolean remission and a sustained period of remission were found to be associated with a better chance of DFR [84]. This suggests that deep clinical remission (likely absence of inflammation at the molecular level) is necessary for successful discontinuation.

Successful TNFi discontinuation has been associated with male gender [70,72], younger age [85], normal body mass index (BMI) [81], not smoking [59], a negative shared epitope [86], longer treatment duration [87] and first-line (vs. delayed) TNFi treatment [72]. The question has been raised whether the type of b-DMARD used for remission induction may affect the chance of DFR. Hashimoto et al. [84] found that using IFX, ADA, and GOL, compared to ETN or CZP, was more advantageous for achieving b-DMARD-free remission in a retrospective registry study of patients discontinuing b-DMARDs. These findings support those found in the POET study, which highlights that those patients who were using a TNFi monoclonal antibody (mainly ADA) were more frequently able to successfully discontinue their TNFi compared to patients who had been using a receptor antagonist (mostly ETN) [79]. The rationale behind this is thought to be due to variations in the mechanism of action and the pharmacokinetic properties of the different agents [88,89]. Hashimoto et al. also demonstrated that no glucocorticoid use at the time of b-DMARD discontinuation was important for the achievement of DFR [84]. The importance of tapering glucocorticoids prior to b-DMARD discontinuation has been recommended by the EULAR guidelines [90]. Patients treated with TCZ in the DREAM study had high rates of relapse following discontinuation compared to that described for TNFis [76].

### 6.2. Patient Reported Outcomes (PRO) Measures

Lower PRO scores at baseline have been shown to be associated with a better chance of successful maintenance of remission after b-DMARD withdrawal to achieve DFR. Good baseline functional status at ADA discontinuation (assessed by standardized patient questionnaires) has been shown to be predictive of low disease activity in the OPTIMA trial [63] and worsening functional disability has been shown to be associated with disease flare [25]. These findings are supported by the AVERT study [75], where lower baseline HAQ (health assessment questionnaire) was associated with successful DFR. The BeST study also found that lower HAQ score was associated with sustained DFR remission in their DAS-guided tapering cohort [45].

### 6.3. Imaging Variables

Musculoskeletal US has been shown to be a reliable method of predicting relapse in patients in clinical remission [91]; therefore, there is interest in using this tool to identify patients who may be able to taper or discontinue biologic therapy. Studies have revealed that the presence of synovitis (measured using power Doppler (PD) assessment) could predict failure of b-DMARD tapering for RA patients in clinical remission and that PD was a good predictor of disease flare within six months of tapering [92,93,94]. Additionally, grey scale synovial hypertrophy (a measure of damage secondary to prior inflammation) is predictive of flare. Furthermore, El Miedany et al. [25] concluded that US was superior to DAS28 in predicting relapse for RA patients in remission, and both PD synovitis and synovial hypertrophy were independent predictors of relapse. Interestingly, Alivernini et al. [95] found that PD synovitis correlated with the histological characteristics of synovial tissue in established RA patients, thus suggesting that US, when combined with clinical remission criteria, could be a useful tool to identify patients likely to achieve DFR.

In light of these findings, US assessment, either alone or in combination with clinical measures, could evaluate remission more objectively and could help identify the best candidates for b-DMARD tapering, towards DFR [72]. MRI findings, e.g., bone marrow oedema, can also identify subclinical synovitis in RA remission and has been shown to be predictive of structural progression [19,96,97,98].

### 6.4. Immunological Variables

To date, the best studied predictor of relapse on tapering/discontinuation of b-DMARDs is ACPA positivity. This indicated higher risk of relapse following dose reduction and lower chances of maintaining remission status [52,70,99,100].

IGM-RF was also associated with a reduced chance of TNFi-free remission [25,69,70,76,101].

Immune dysregulation is key to the pathogenesis of RA. Inflammation has a direct effect on T-cell differentiation and promotes the differentiation and proliferation of naïve CD4+ T-cells towards an abnormal phenotype. Characteristically, there is dysregulation of pro-inflammatory CD4+ T-helper cell subsets (naïve, regulatory (Treg) and inflammation-related cells (IRC)) [102,103]. Abnormalities in T-cell subsets have been found across the spectrum of RA and can predict progression, from ‘at-risk’ individuals to evolving RA and those in clinical remission [31]. In a study comparing the characteristics of 47 patients undergoing TNFi tapering, Saleem et al. [72] found that sustained remission was associated with T-cell subset immunological abnormalities. Patients who sustained remission for 24 months presented a higher frequency (%) of naïve T-cells and lower frequency of IRCs. Furthermore, the frequency of Treg cells was higher in the sustained remission group. These proportions were different for the patients receiving early, aggressive treatment compared to delayed treatment, for whom Treg frequency was higher.

### 6.5. Serum Biomarkers and Multi-Biomarker Assays

Multi-biomarker disease activity (MDBA) assays, developed to identify subclinical inflammation at the molecular level, have been investigated in several studies of RA patients in clinical remission. In general, studies have found that MDBA scores may be elevated in patients deemed to be in remission according to conventional clinical definitions [104,105,106,107]. These patients were also found to have a higher risk of structural joint damage [9,107].

One such score involves a total of 12 inflammation parameters, including markers linked to the acute phase [104]. It was initially developed and validated to correlate with the DAS28CRP score. Two studies have demonstrated that the score is better at predicting radiological progression than the DAS28CRP score [105,108]. In patients with high baseline MBDA scores at discontinuation of TNFi in the POET study, discontinuation may have allowed a recurrence of residual subclinical inflammation and the need to recommence TNFi treatment [79].

In the RETRO study [70], the MBDA score could predict the relapse of more than 80% if combined with ACPA testing (if both were positive). This highlights that both inflammation and autoimmunity are key players in the risk of flare in RA patients undertaking b-DMARD reduction. Of note, despite MDBA scores being higher at baseline and flare in the tapering groups, there was no difference between sustained remission and flare in the non-tapering group. Conversely, the DRESS [68] study did not find any association between MDBA score or ACPA status on flare outcome in the tapering group but did in the stable therapy group. This may be due to the fact that the study was evaluating patients in low disease activity (DAS28 < 3.2) as opposed to remission. Nishimoto et al. provide additional evidence to support the predictive value of serological biomarkers for discontinuing b-DMARDs [76].

Collectively, these findings indicate that evaluating subclinical inflammation using serum biomarkers may be a useful tool to determine risk of flare/high risk candidates in whom tapering or discontinuation of therapy should not be initiated. Validation of this work is required.

### 6.6. Deep/Multi-Level Remission

As previously described, it is thought that achieving deep clinical remission is required to facilitate DFR [7].

Building on this, Gul et al. [109] aimed to define remission more precisely using a multi-dimensional model of remission using clinical, US and T-cell subset measures (for patients treated with either cs or b-DMARDs). In this cross-sectional study, considerable heterogeneity of DAS28 remission was observed with respect to these characteristics, with some patients showing evidence of high inflammatory markers and joint counts, evidence of synovitis on PD US and persistent T-cell subset abnormalities (which should not be present in remission). Definitions for clinical, US and T-cell subset remission were created and the achievement of all three was thought to represent a state of complete remission (multi-dimensional remission (MDR)). Out of approximately 200 patients, only 30% satisfied the criteria for MDR. These patients were found to have lower PRO scores. Further work has resulted in the development of a predictive model for successful tapering (towards DFR) of cs-DMARDs and work is underway to replicate this in a cohort of patients undergoing tapering and discontinuation of b-DMARDs [55]. This could help inform tapering strategies in clinical practice.

## 7. DFR Summary and Clinical Guidance

This article has summarized the latest evidence regarding a highly topical area within Rheumatology. Clinical trial data for each therapeutic group have been summarized to provide an overview of the evidence that provides us with clinical guidance to manage patients in remission. Importantly it has been demonstrated that DFR is possible in a proportion of RA patients achieving clinically defined remission (both on cs and b-DMARDs). It must be highlighted that most studies in the literature focus mainly on b-DMARDs, with significantly few studies for cs-DMARDs in comparison.

Associations with/predictors of DFR have been identified, which are promising, but prospective studies are lacking. Most importantly, DFR appears to be more successful with early and deep (molecular) remission induction, followed by gradual tapering of therapy. Thus, defining remission more precisely using objective markers of inflammation is important to be able to identify patients who can successfully stop therapy. This is more favorable than current disease activity-guided tapering methods, which are largely based on trial and error. For patients taking combination therapy, discontinuing the b-DMARD followed by the cs-DMARD appears to be the most logical approach. Modelling of predictors for sustained DFR is underway in some centers, although their application in the clinical setting is not yet standard of care. It is hoped that these could inform future decisions to discontinue treatment in RA patients.

In practice, a pragmatic approach to reducing therapy must be taken, with patients given the choice to taper or continue their treatment (as per EULAR guidelines). The risks, i.e., flare and disease progression, as well as the benefits must be explained, and a discussion must take place regarding treatment target when first diagnosed. Strict disease activity monitoring and patient education re: management and recognition of flare will also be crucial to the success of DFR.

## Figures and Tables

**Table 1:** cs-DMARD DFR remission studies.

Study	Design	Authors	*n*	Treatment/Intervention	RA Disease Duration	Remission Criteria	%DFR Remission	DFR-Predicting Factors	Follow Up Period
Can disease-modifying anti-rheumatic drugs be discontinued in long standing rheumatoid arthritis? A 15-year follow-up	Observational	Tiippana et al., 2010	70	Single or combination Cs-DMARDS tapered	Early RA	5/6 ARA criteria fulfilled.	16%	N/A	15 years
Prevalence and predictive factors for sustained disease-modifying antirheumatic drug-free remission in rheumatoid arthritis: results from two large early arthritis cohorts	Observational	van der Woude et al., 2009	Leiden EAC cohort: 454 British EAC Cohort: 895	Single or combination Cs-DMARDS tapered (MTX/SSZ/HcQ)	Early RA	Had to fulfil 3 criteria: (1) No current use of DMARDs/corticosteroids, (2) No swollen joints, and (3) Classification as DMARD-free remission by the patient’s rheumatologist.	Leiden EAC cohort: 15% British EAC Cohort: 9.4%	Absence of autoantibodies ((ACPA and IgM-RF) and short symptom duration at presentation	Minimum of 1 year after discontinuation of DMARD therapy
KIMERA	Observational	Jung et al., 2020	234	Single or combination therapy with cs DMARDs; methotrexate (MTX)/sulfasalazine combined with high-dose glucocorticoid; MTX combined with TNF-inhibitors tapered	Early RA	(1) Non-use of cs or bDMARDs and glucocorticoids, (2) DAS28 <2.6, and (3) no swollen joints.	46.1%	Early RA and lower disease activity (DAS28 <2.26) at csDMARD withdrawal	48 months
Randomized placebo-controlled study of stopping second-line drugs in RA	RCT	Ten Wolde et al., 1996	285	Placebo or withdrawal of at least one 2nd line cs-DMARD (chloroquine, HCQ, gold, d-penicillamine, SSZ, AZA or MTX)	Established RA. Median duration 8–9 years.	5/6 ARA criteria fulfilled	62%	Lower maintenance dose of second line drug and absence of RF	52 weeks
D-penicillamine withdrawal in rheumatoid arthritis	Double blind RCT	Ahern et al., 1984	38	Tapering of d-penicillamine	Established RA (6–11 years)	5/6 ARA criteria fulfilled	21%	None	12 months
BeST	Multi center randomized single blind trial	Markusse et al., 2015	508	MTX/combination cs DMARD/ combination cs-DMARD +prednisolone/combination cs DMARD with MTX and Infliximab	Early disease (symptom duration < 2 years)	DAS44 <1.6	14%	Absence of ACPA and using MTX rather than SSZ as the last csDMARD before withdrawal	10 years
tREACH	RCT	Kuijper et al., 2016	281	Triple cs-DMARD (MTX, SSZ and HCQ) with glucocorticoid bridging or MTX monotherapy with glucocorticoid bridging TNFi and MTX if the DAS28 was >2.4.	Early RA	DAS28 <1.6	2.4%	N/A	2 year
IMPROVED	RCT	Heimans et al., 2016	610	MTX and prednisolone, then tapered	Early RA or Undifferentiated arthritis	DAS44 <1.6	21%	Absence of ACPA	2 year
BioRRA	Interventional cohort study	Baker et al., 2019	44	Cessation of cs-DMARDs	Established RA	DAS28-CRP < 2.4	48%	Absence of RF, shorter time from diagnosis to starting first DMARD, shorter symptom duration at time of diagnosis, longer disease duration fulfilment of ACR/EULAR Boolean remission criteria and longer time since last DMARD change Absence of genes within peripheral CD4+ T cells; *FAM102B* and *ENSG00000227070* Presence of gene within peripheral CD4+ T cells: *ENSG00000228010*	6 months

**Table 2:** Biologic DFR remission studies.

Study	Design	Authors	*n*	Treatment/Intervention *Drug Withdrawn in Italics*	RA Disease Duration	Remission Criteria	%DFR Remission in Biologic Treatment Arm	DFR Predicting Factors	Follow Up Period
IVEA	Double blind RCT	Quinn MQ et al., 2006	20	1. *Infliximab* + MTX 2. MTX	6 months	DAS28	70	-	12 months
BeSt	RCT	van den Broek M et al., 2011	128	4th study arm: Combination with *infliximab*	23 months	DAS44	56	Lower baseline HAQ ACPA negative Lower baseline disease activity Younger age Non-smoker	24 months
IDEA	Double blind RCT	Nam JL et al., 2014	112	1. *Infliximab* +MTX 2. MTX + single dose IV methylprednisolone	78 weeks	DAS44	76%	-	78 weeks
HONOR	Open label non randomized	Yamaguchi A et al., 2020	52	*Adalimumab*	7 years	DAS28	21	A baseline DAS28 of <2.22 or <1.98 Shorter disease duration	60 months
RRR *	Observational	Tanaka Y et al., 2010	114	*Infliximab*	6 years	LDA	55	A baseline DAS28 of <2.22 or <1.98	12 months
OPTIMA	RCT	Smolen J et al., 2013	1032	*Adalimumab + MTX*	≤12 months	DAS28	66%	Good baseline functional status	52 weeks
PRIZE	Double blind RCT	Emery P et al., 2014	306	1. *½ dose Etanercept* + MTX 2. Placebo + MTX 3. Placebo alone	≤12 months	DAS2	23–40%	-	39 weeks
CERTAIN	Double blind RCT	Smolen J et al., 2015	194	1. *Certolizumab* + MTX 2. Placebo	6 months–10 years	CDAI	18.8%	-	52 weeks
Patients with RA in remission on TNF blockers: when and in whom can TNF blocker therapy be stopped?	Observational	Saleem et al., 2011	47	*TNFi (Various) +* MTX 1. Initial therapy 2. Delayed therapy	12 months	DAS28	59%15%	Male gender First line TNFi Shorter disease duration Higher and naïve T-cells and fewer IRCs at baseline	24 months
EMPIRE	Double blind RCT	Nam et al., 2013	110	1. *Etanercept* + MTX 2. MTX + placebo	≤3 months	DAS28	28.1%	Starting TNFi earlier in disease course	52 weeks
TARA	Single blind RCT	Van Mulligen et al., 2020	189 94 DMARD 95 TNFi	*TNFi or csDMARD (Various)* *1. csDMARD taper first* *2. TNFi taper first*	Not stated	DAS44	15%	-	24 months
AVERT	Double blind RCT	Emery P et al., 2015	351	*Abatacept + MTX*	<1 year	DAS28	15%	Lower baseline PRO scores	18 months
DREAM	Observational	Nishimoto N et al., 2014	187	*Tocilizumab*	7.8 years	LDA	9%	Lower multi-biomarker assay scores (serological) RF negative	12 months
ACT RAY	RCT	Huizinga TW et al., 2015	556	*Tocilizumab*	8 years	DAS28	6%	Shorter disease duration, few/absent erosions	12 months
RETRO	RCT	Haschka J et al., 2016	101	*Various*	NK	DAS28	48.1%	ACPA negative Lower baseline disease activity Male gender Lower multi-biomarker assay scores (serological) RF negative	12 months
PredictRA	Double blind RCT	Emery et al., 2020	122	*Adalimumab taper* vs. *withdrawal*	Mean 12.9 years	DAS28	55% (withdrawal arm)	-	36 weeks
ANSWER	Cohort	Hashimoto et al., 2018	181	*Various*	NK	DAS28	21.5%	Boolean remission at baseline Sustained remission period No glucocorticoid use at time of discontinuation TNFi discontinuation (vs. other b-DMARD)	12 months

* NK = not known.
